# A novel strategy for l-arginine production in engineered *Escherichia coli*

**DOI:** 10.1186/s12934-023-02145-8

**Published:** 2023-07-26

**Authors:** Mengzhen Nie, Jingyu Wang, Kechun Zhang

**Affiliations:** 1 Zhejiang University, Hangzhou, 310027 Zhejiang China; 2 Center of Synthetic Biology and Integrated Bioengineering, School of Engineering, Westlake University, Hangzhou, 310030 Zhejiang China

**Keywords:** *Escherichia coli*, l-Arginine, n-acetylglutamate, Metabolic engineering

## Abstract

**Background:**

l-arginine is an important amino acid with applications in diverse industrial and pharmaceutical fields. n-acetylglutamate, synthesized from l-glutamate and acetyl-CoA, is a precursor of the l-arginine biosynthetic branch in microorganisms. The enzyme that produces n-acetylglutamate, n-acetylglutamate synthase, is allosterically inhibited by l-arginine. l-glutamate, as a central metabolite, provides carbon backbone for diverse biological compounds besides l-arginine. When glucose is the sole carbon source, the theoretical maximum carbon yield towards l-arginine is 96.7%, but the experimental highest yield was 51%. The gap of l-arginine yield indicates the regulation complexity of carbon flux and energy during the l-arginine biosynthesis. Besides endogenous biosynthesis, n-acetylglutamate, the key precursor of l-arginine, can be obtained by chemical acylation of l-glutamate with a high yield of 98%. To achieve high-yield production of l-arginine, we demonstrated a novel approach by directly feeding precursor n-acetylglutamate to engineered *Escherichia coli*.

**Results:**

We reported a new approach for the high yield of l-arginine production in *E. coli.* Gene *argA* encoding n-acetylglutamate synthase was deleted to disable endogenous biosynthesis of n-acetylglutamate. The feasibility of external n-acetylglutamate towards l-arginine was verified via growth assay in *argA*^−^ strain. To improve l-arginine production, *astA* encoding arginine n-succinyltransferase, *speF* encoding ornithine decarboxylase, *speB* encoding agmatinase, and *argR* encoding an arginine responsive repressor protein were disrupted. Based on overexpression of *argDGI, argCBH* operons*,* encoding enzymes of the l-arginine biosynthetic pathway, ~ 4 g/L l-arginine was produced in shake flask fermentation, resulting in a yield of 0.99 mol l-arginine/mol n-acetylglutamate. This strain was further engineered for the co-production of l-arginine and pyruvate by removing genes *adhE, ldhA, poxB, pflB,* and *aceE,* encoding enzymes involved in the conversion and degradation of pyruvate*.* The resulting strain was shown to produce 4 g/L l-arginine and 11.3 g/L pyruvate in shake flask fermentation.

**Conclusions:**

Here, we developed a novel approach to avoid the strict regulation of l-arginine on ArgA and overcome the metabolism complexity in the l-arginine biosynthesis pathway. We achieve a high yield of l-arginine production from n-acetylglutamate in *E. coli*. Co-production pyruvate and l-arginine was used as an example to increase the utilization of input carbon sources.

**Supplementary Information:**

The online version contains supplementary material available at 10.1186/s12934-023-02145-8.

## Background

l-arginine is a useful amino acid that plays a crucial role in maintaining normal biological functions, including stimulating the secretion of growth hormones [1], promoting wound healing [2], and relaxing blood vessels as a precursor of nitric oxide [3]. l-arginine is also commonly used in pharmaceuticals [4, 5], cosmetics [6], and dietary supplements [7]. Given the widespread applications and important demands of l-arginine, there is a need to develop efficient methods, especially to replace traditional natural extraction and petrochemical synthesis methods to produce l-arginine. In recent years, various new strategies based on genetic engineering have been proposed to increase the bio-production level of l-arginine, including random mutagenesis [8], removing competing pathways [9], strengthening upstream pathways for increasing precursor pool [10, 11], engineering transporters [12] and fine-tuning expression levels [13, 14]. For example, by optimizing NADPH level and precisely regulating the flux of the l-arginine biosynthesis pathway, a *C. glutamicum* strain has been constructed and demonstrated to be able to achieve a yield of 0.4 g l-arginine per gram carbon source during fermentation [15]. Recently, l-arginine production from glucose with a yield of 0.51 g/g glucose has been achieved by fine-reprogramming of the *E. coli* central metabolic network and screening random mutagenesis with the help of the transcriptional factor-based biosensor [16]. Nevertheless, when glucose is the sole carbon source, the theoretical maximum carbon yield towards l-arginine is 96.7% (by weight) [17], and the reported experimental highest yield was 51% [16], implying significant carbon and energy loss during l-arginine biosynthesis pathway. There could be a new approach to improve the metabolic flux yield in the l-arginine biosynthesis pathway.

From l-glutamate to l-arginine, the key precursor in the branch pathway is n-acetylglutamate, which is synthesized from acetyl-CoA and l-glutamate [18, 19]. When glucose is used as the sole carbon source, acetyl-CoA is primarily supplied by pyruvate decarboxylation under aerobic conditions. However, significant amounts of acetyl-CoA would be consumed in the active tricarboxylic acid (TCA) cycle, along with both carbon and energy loss [20]. l-glutamate biosynthesis requires acetyl-CoA, oxaloacetate, and NADH [21]. In addition, l-glutamate as a central metabolite, not only participates in nitrogen metabolism with glutamine [22] but also provides carbon backbone for diverse biological compounds besides l-arginine [23]. Therefore, the complex flux distribution of acetyl-CoA and l-glutamate could lead to an imbalance of metabolic cofactor and carbon flux, which may be one of the reasons restricting the practical carbon yield of l-arginine production.

While the first intermediate, n-acetylglutamate, in the branch, is subjected to complicated regulation in biosynthesis, the chemical conversion of l-glutamate to n-acetylglutamate is simple and high-yield. n-acetylglutamate can be obtained with a yield of 98% by mixing acetic anhydride, l-glutamate, and water into ultrasonic waves for 10 mins at ambient temperature [24, 25]. In the past decades, the improvement in l-glutamate fermentation technology leads to a continued decline in its price [26], on the other hand, acetic anhydride is a bulk chemical with a price of ~$0.5/kg [27]. Therefore, we envision this hybrid approach is economically feasible to replace the endogenous biosynthesis of n-acetylglutamate and potentially overcome the loss of carbon yield in the l-arginine biosynthesis pathway.

**Fig. 1:**
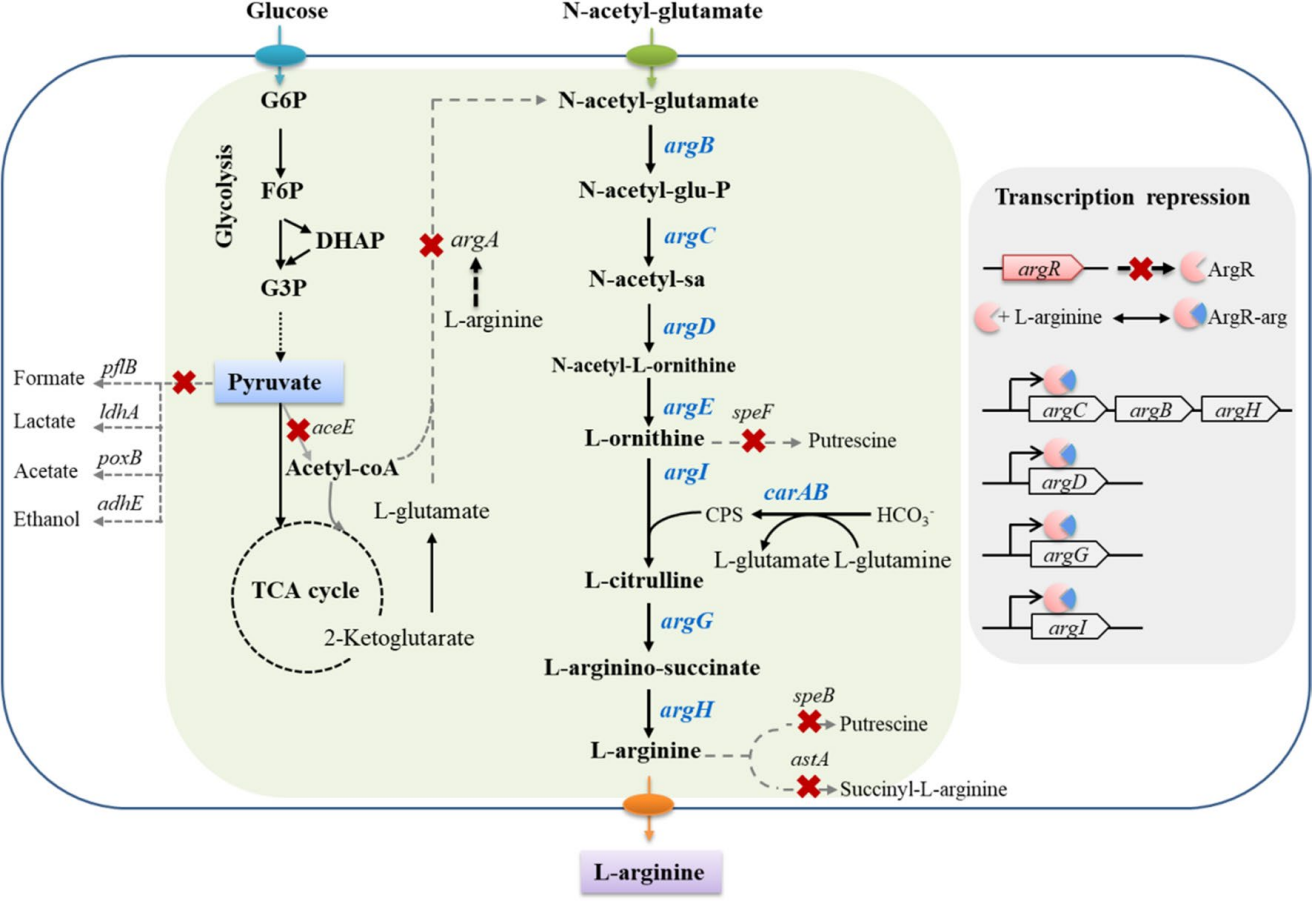
Major metabolic pathways associated with l-arginine biosynthesis in *E. coli* and metabolic engineering approaches applied to overproduce l-arginine. Dotted gray arrows with  indicate the deletion of the relevant genes. Black thick arrows indicate increased fluxes by directly overexpressing the corresponding genes. Black dashes indicate the negative feedback inhibition mechanisms. The role of ArgR repressor regulation on l-arginine production is shown in the gray box. Abbreviations: *G6P* glucose 6-phosphate, *F6P* fructose 6-phosphate, *DHAP* dihydroxyacetone phosphate, *G3P* glyceraldehyde 3-phosphate, *n-acetyl-glu-P* n-acetylglutamate-phosphate, *n-acetyl-sa* n-acetylglutamate-semialdehyde, *CPS* carbamoyl phosphate

In this study, we proposed an innovative hybrid strategy to achieve a high yield of l-arginine biosynthesis from n-acetylglutamate in engineered *E. coli*. n-acetylglutamate was synthesized from the chemical acylation of l-glutamate with high yield. The specific details of the strategy are shown in Fig. 1. We knocked out gene *argA* to block internal l-glutamate acylation and externally fed n-acetylglutamate as the precursor to produce l-arginine. With the external n-acetylglutamate supply, *argA*^−^ strain grows well in growth assay. Then, l-arginine degradative pathways (encoded by genes *astA, speB, speF*) and transcriptional regulator ArgR were removed, and the phenotype of the resultant strains was investigated as well. Furthermore, the genes involved in l-arginine biosynthesis were overexpressed to strengthen l-arginine production. The engineered strains were validated by shaking flask fermentation under controlled conditions. Taken together, we accomplished ~ 4 g/L l-arginine production in shaking flask fermentation of strain N11 with n-acetylglutamate feeding, which resulted in a yield of 99% of the theoretical maximum (the theoretical yield is 1 mol l-arginine per mole n-acetyl-glutamate). Moreover, to avoid the waste of glucose cosubstrate, we simultaneously convert glucose to valuable product pyruvate besides converting n-acetylglutamate to l-arginine. We disrupted the conversion and degradation of pyruvate by removing genes *adhE, ldhA, poxB, pflB,* and *aceE* in strain N11 background, and the resulting strain N12 achieved the accumulation of 4 g/L l-arginine and 11.3 g/L pyruvate simultaneously.

## Results and discussion

### n-acetylglutamate could support growth of *E. coli* Δ*argA*

In the native l-arginine biosynthetic pathway of *E. coli,* the allosteric enzyme ArgA, n-acetylglutamate synthase, the key rate-limiting enzyme in l-arginine synthesis pathway, is inhibited by intracellular l-arginine, n-acetylglutamate and  acetyl-CoA [28–30]. In a previous study, homologous overexpression of gene *cg3035*, screened from a genomic library of *C. glutamicum*, was able to complement the *E. coli ΔargA* mutant and n-acetylglutamate was the only detectable metabolite in culture supernatant of this mutant [31]. There were previously no reports on rescuing the growth of *E. coli ΔargA* mutant via direct n-acetylglutamate feeding. To address this issue, gene *argA* was knocked out in wild-type *E. coli* BW25113 to disable n-acetylglutamate endogenous biosynthesis and obtain an l-arginine auxotrophic strain N1 (Table 1). The growth ability of strain N1 was evaluated using the M9-based medium in a shake-flask with or without exogenous l-arginine or n-acetylglutamate supply.

**Table 1:** Strains and plasmids used in this study

Strains/plasmids	Phenotype	Source
Strains		
BW25113	*Δ(araD-araB)567ΔlacZ4787(::rrnB-3) ΔlacZ4787(::rrnB-3) Δ(rhaD-rhaB)568 hsdR514*, starting strain	CGSC
*E. coli* DH5α	Host for plasmid construction	This study
N1	BW25113 *ΔargA*	This study
N2	BW25113 *ΔargA ΔspeF ΔspeB*	This study
N3	BW25113 *ΔargA ΔspeF ΔspeB ΔastA*	This study
N4	BW25113 *ΔargA ΔspeF ΔspeB ΔastA ΔargR*	This study
N5	BW25113 [pZE]	This study
N6	N2 [p-AGR-1]	This study
N7	N3 [p-AGR-1]	This study
N8	N4 [p-AGR-1]	This study
N9	N4 [p-AGR-2]	This study
N10	N4 [pZE]	
N11	N4 [p-AGR-3, p-AGR-4]	This study
N12	N4 *ΔldhA ΔadhE ΔaceE ΔpoxB ΔpflB* [p-AGR-3, p-AGR-4]	This study
Plasmids		This study
p-AGR-1^a^	pZE-*P*_*LlacO1*_*-argI-argG-argH*	This study
p-AGR-2^a^	pZE-*P*_*LlacO1*_*-carAB-argI-argG-argH*	This study
p-AGR-3^a^	pZE-*P*_*LlacO1*_*-argCBH*	This study
p-AGR-4^a^	pZA-*P*_*LlacO1*_*-argD-argG-argI*	This study
pCP20	FLP recombinase expression	CGSC

^a^The isopropyl-β-D-thio-galactoside (IPTG) was required to induce the overexpression of introduced genes in plasmids

**Fig. 2:**
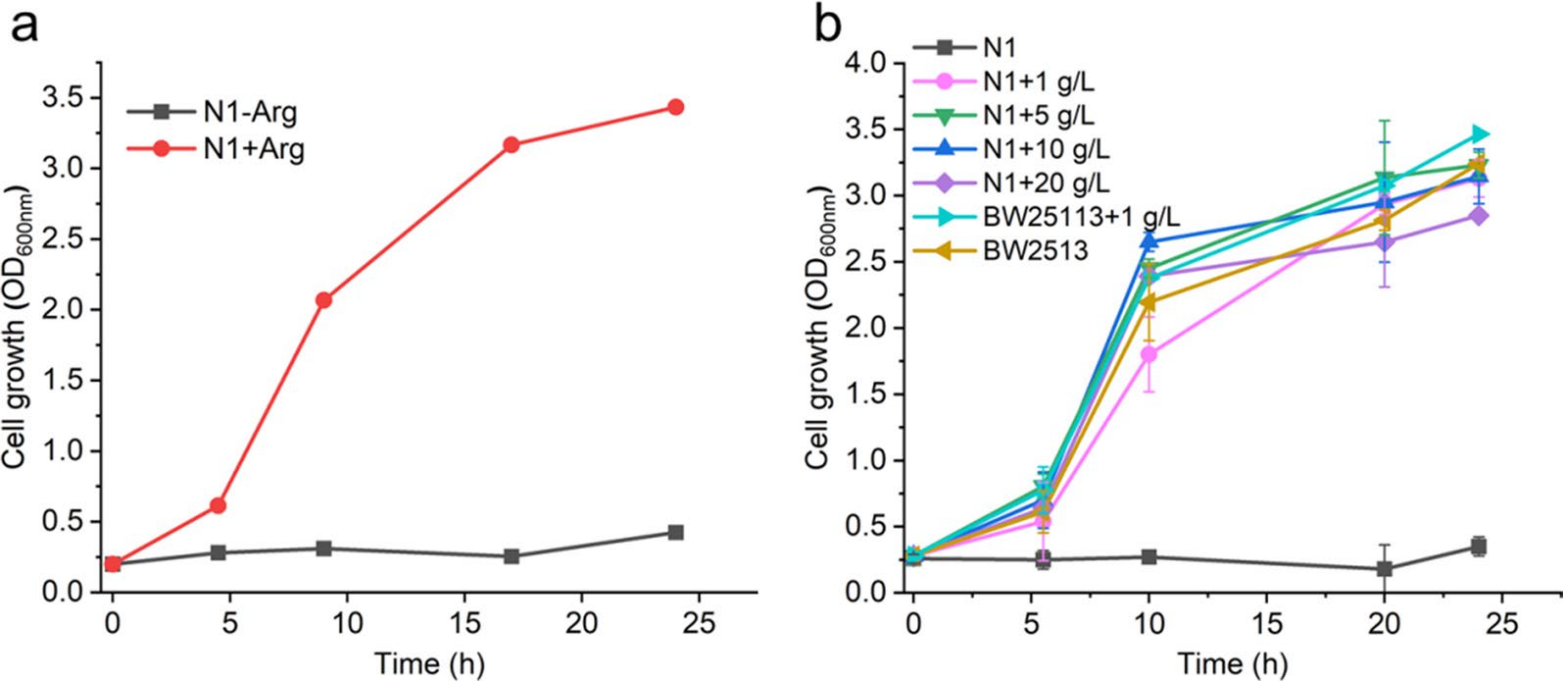
Effects of gene *argA* deletion on cell growth. **a** Growth curves of strain N1 (*E. coli ΔargA*) in M9 minimal medium supplemented with 5 g/L glucose (without yeast extract), -Arg means the absence of l-arginine. + Arg means the presence of l-arginine. **b** Growth curves of strain N1 and BW25113 in M9 minimal medium added with different concentrations of n-acetylglutamate (0 g/L, 1 g/L, 5 g/L, 10 g/L, 20 g/L). Error bars are the standard deviation for three independent experiments

Strain N1 could not grow in the minimal medium containing glucose owing to the disruption of natural l-arginine biosynthesis, and the cells required an exogenous supply of l-arginine to grow (Fig. 2a), which was consistent with the previous study that no growth was observed without l-arginine supplementation in M9 medium using a similar strategy [31]. Then, strain N1 was cultured in media with different n-acetylglutamate levels (0, 1, 5, 10, and 20 g/L) to further evaluate the correlation between the cell growth and n-acetylglutamate supply (Fig. 2b). With 1 g/L of n-acetylglutamate supply, the growth of strain N1 was restored (OD_600_ increased to 1.8 within 10 h, and up to 3.1 after 24 h), and showed a similar growth pattern to that of wild-type *E. coli* BW25113. With feeding of 5 g/L of n-acetylglutamate, N1 showed more robust growth (OD_600_ reached 2.7 within 10 h, and saturated at 3.3). Further higher n-acetylglutamate supply (10 g/L and 20 g/L) led to a growth pattern that was  very similar to that of 5 g/L n-acetylglutamate supplied. These results indicated that external n-acetylglutamate could be incorporated into the intracellular l-arginine biosynthesis pathway and then successfully rescued the cell growth of l-arginine auxotrophic strain N1. It's worth noting that the external high n-acetylglutamate pool did not repress cell growth, implying the l-arginine bio-production could start from high input flux of n-acetylglutamate without the issue that feedback inhibition of ArgA by l-arginine in the typical total l-arginine biosynthesis pathway.

### Deletion of genes encoding enzymes involved in l-arginine degradation and regulator involved in l-arginine biosynthesis

Blocking the degrading and competing pathway is a useful strategy in metabolic engineering to achieve a higher accumulation of target metabolites. In *E. coli*, l-arginine and its precursor l-ornithine can be decomposed into putrescine, urea, or succinate [32, 33]. Therefore, we disrupted genes *speFB* and *astA* in strain N1 sequentially to obtain strain N2 and strain N3 (Table 1). Furthermore, gene *argR*, encoding the regulator of the l-arginine biosynthesis operon (*arg*), was knocked-out due to ArgR repressing the expression of many enzymes of the l-arginine biosynthesis pathway [34, 35], resulting in strain N4 (Table 1). In *E. coli*, l-ornithine was an intermediate in the l-arginine biosynthetic pathway [11, 36]. Therefore, the feasibility of l-arginine production was explored by supplying the precursor l-ornithine in our case.

**Fig. 3:**
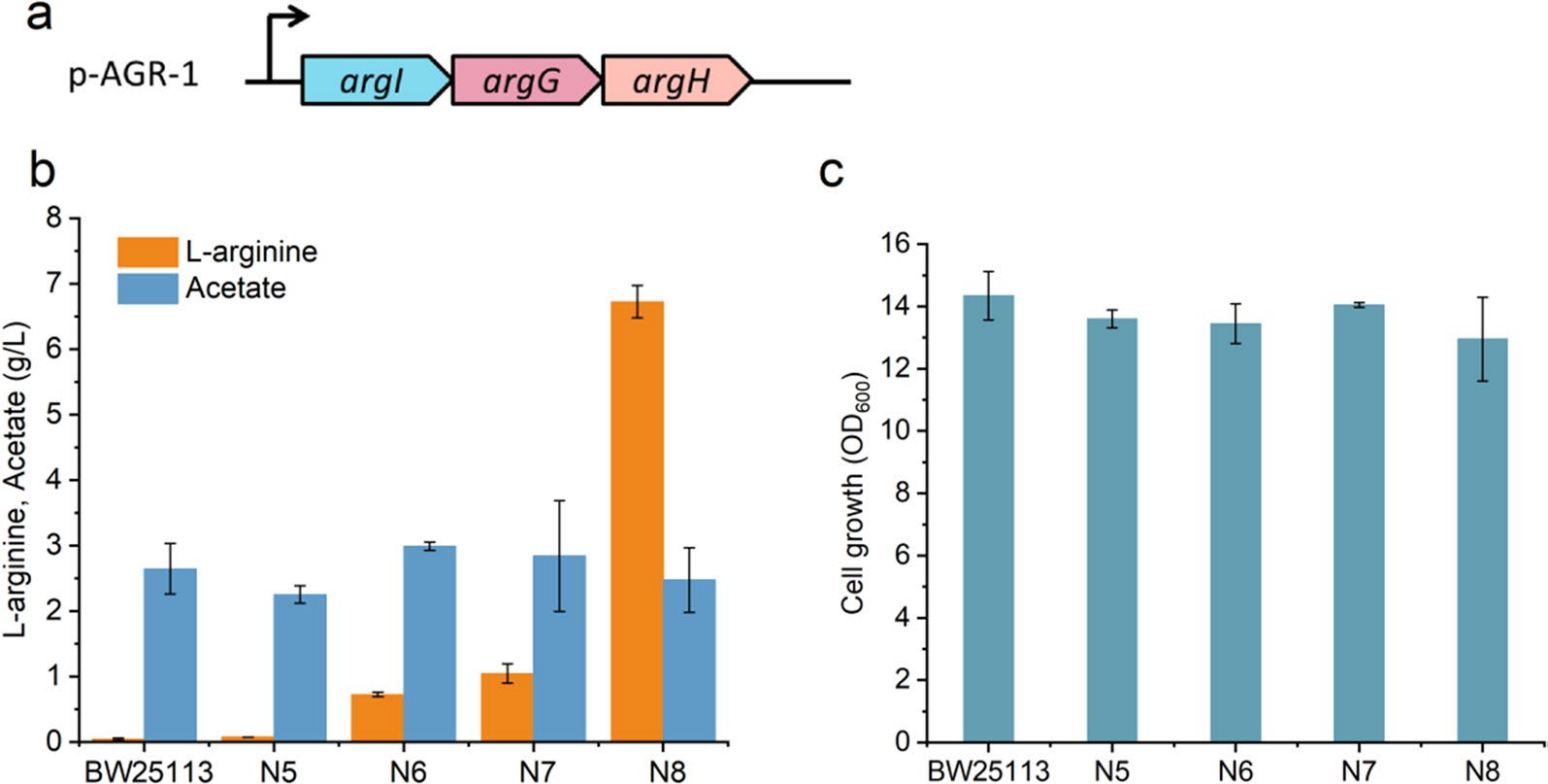
Effects of removing the l-arginine degradation pathway and transcriptional repressor ArgR on l-arginine production from glucose and l-ornithine. **a** Plasmid p-AGR-1 was constructed for l-arginine overproduction. **b** l-arginine production and acetate formation of strains BW25113 and N5-N8. **c** Cell growth of strains BW25113 and N5-N8. N5: BW25113 with empty plasmid pZE; N6: BW25113 *ΔargA ΔspeF ΔspeB,* overexpressionof *argIGH*; N7: BW25113 *ΔargA ΔspeF ΔspeB ΔastA,* overexpression of *argIGH*; N8: BW25113 *ΔargA ΔspeF ΔspeB ΔastA ΔargR,* overexpression of *argIGH*. Error bars are the standard deviation for three independent experiments

**Fig. 4:**
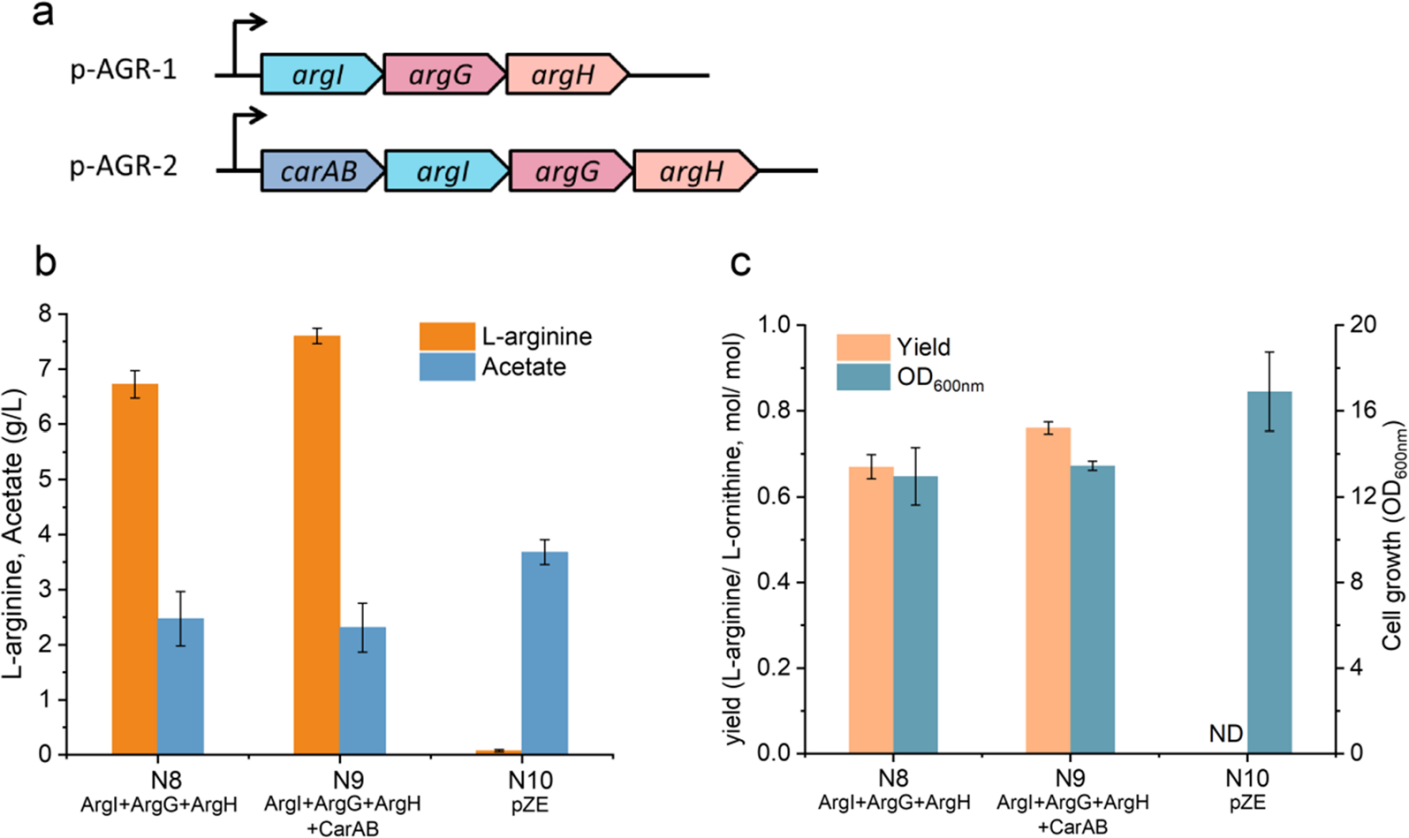
Effects of enhancing the carbamoyl phosphate supply on l-arginine production from glucose and l-ornithine. **a** Plasmids p-AGR-1 and p-AGR-2 were constructed for l-arginine overproduction. **b** l-arginine production and acetate formation of strains N8-N10. **c** Cell growth and l-arginine yield from l-ornithine of strains N8-N10. N8: BW25113 *ΔargA ΔspeF ΔspeB ΔastA ΔargR*, overexpression of *argIGH*; N9: BW25113 *ΔargA ΔspeF ΔspeB ΔastA ΔargR*, overexpression of *argIGH, carAB*; and N10: BW25113 *ΔargA ΔspeF ΔspeB ΔastA ΔargR*, with empty plasmid pZE. Error bars are the standard deviation for three independent experiments

In order to increase the conversion of l-ornithine to l-arginine, a high copy plasmid containing gene cluster *argIGH* (p-AGR-1, shown in Fig. 3a)*,* encoding enzymes for converting l-ornithine to l-arginine, was constructed and transformed into strains N2-N4 respectively to obtain strains N6, N7 and N8 (Table 1). The l-arginine production performance was evaluated in a shake-flask with 10 g/L l-ornithine feeding. It was clear that a significant difference in the l-arginine titer was detected at the end of the fermentation in these different strains (Fig. 3b). The parental strain BW25113, without engineering or transformed with empty plasmid pZE (strain N5, Table 1), both have very low titers of l-arginine (0.07 g/L, column #1 and 0.09 g/L column #3 in Fig. 3b), similar to previous studies [16, 37]. The titers of strain N6 and strain N7 slightly improved to 0.7 g/L and 0.9 g/L (columns #5 and #7 in Fig. 3b), respectively. However, these titers were still very low and there were large quantities of l-ornithine remaining in the fermentation culture (data not shown) [38]. The highest titer of 6.7 g/L (column #9 in Fig. 3b) l-arginine was produced in strain N8 with a yield of 0.69 mol l-arginine/mol input l-ornithine (column #1 in Fig. 4c) when the same media conditions were applied, which was a profoundly over sixfold increase compared with that in strains N6-N7. The substantial increase in l-arginine production of strain N8 confirmed that the strong negative feedback regulation of l-arginine responsive transcriptional repressor ArgR may be an important factor leading to the low yield of l-arginine biosynthesis in strains N6 and N7. The expression of genes involved in l-arginine synthesis in *E. coli* is regulated by protein ArgR, which inhibits the synthesis of l-arginine when intracellular l-arginine concentration increases, similar to previous reports [16, 39].

In addition, in both cases the only significant byproduct observed was acetate, which was produced at a titer of 2.6 g/L, 2.3 g/L, 3 g/L, 2.8 g/L, and 2.5 g/L, respectively, suggesting that glucose, in addition to supporting growth, partially overflowed toward acetate during the l-arginine biosynthesis process. This is consistent with a previous report, where an accumulation of acetate overflow was detected when growing under high glucose consumption [33]. Acetate overflow occurs when the rate of glucose consumption is greater than the ability of the cell to reoxidize the reduced equivalents generated by glycolysis (i.e. NAD(P)H) [40]. Concentration-limited feeding of glucose could be adopted to reduce byproduct accumulation and increase the efficiency of carbon resource utilization [41]. Finally, the cell growth of strains N6–N8 was not distinctly affected, as shown in Fig. 3c. At the end of fermentation, the OD_600_ of strain N8 was similar to BW25113 (13 for strain N8 vs 14.4 for BW25113), which indicated that the resulting intracellular of l-arginine did not have a significantly negative effect on cell growth.

Strain engineering efforts to improve l-arginine titer were successful. Compared to the wild-type *E. coli* BW25113, the synthetic capability of desired l-arginine in engineered strain N8 was significantly improved (6.7 g/L for strain N8 vs 0.09 g/L for BW25113). These results demonstrated a significant synergistic effect on increasing the accumulation of end-product l-arginine by eliminating the l-arginine degradation pathway and inactivating the ArgR feedback regulator.

### Effect of the carbamoyl phosphate on l-arginine production

We next attempted to promote l-arginine biosynthesis by modulating the metabolic flux of carbamoyl phosphate, which is required for converting l-ornithine to l-citrulline by protein ArgI. Carbamoyl phosphate is synthesized by carbamoyl phosphate synthetase, encoded by genes *carAB* in *E. coli*. Previous studies have shown that the transcription of the *carAB* operon was negatively regulated by the accumulation of l-arginine and pyrimidine nucleotides [42, 43]. To increase the carbamoyl phosphate supply, genes *carAB* were further overexpressed (p-AGR-2, shown in Fig. 4a) based on strain N8, resulting in strain N9 (Table 1). The shake flask fermentation results showed that overexpression of *carAB* could further enhance the l-arginine titer to 7.6 g/L (1.1 × increase compared with the strain N8, column #3 in Fig. 4b), with a yield of 0.76 l-arginine mol/mol l-ornithine (column #3 in Fig. 4c). In the control strain N10 (strain N4 transformed with empty plasmid pZE, Table 1), only 0.05 g/L l-arginine was detected. Furthermore, the growth of strain N9 was not affected, and the OD_600_ reached 13.3 at the end of fermentation (column #4 in Fig. 4c). These results indicated that reinforcing the carbamoyl phosphate supply can indeed enhance the production of l-arginine with l-ornithine supplied, which is consistent with the previous report [44].

These above results indicated that the engineered strain can synthesize l-arginine from exogenous l-ornithine with a high yield. With the downstream optimization of the l-arginine biosynthesis pathway, we moved upstream to efficiently produce l-arginine from n-acetylglutamate in the strain we constructed.

### l-arginine biosynthesis from n-acetylglutamate

**Fig. 5:**
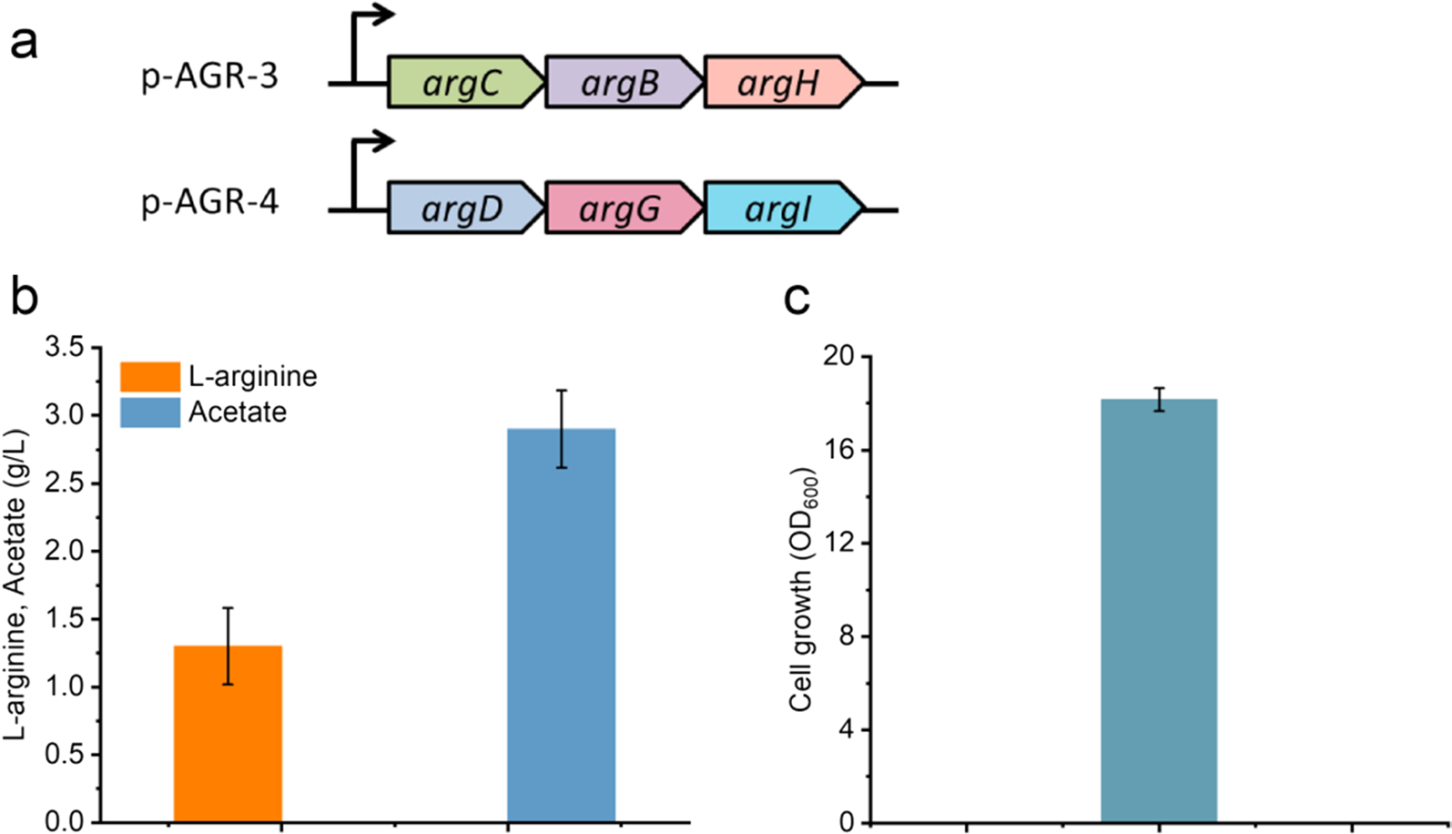
Production of l-arginine in strain N11 from glucose and n-acetylglutamate. **a** Plasmids p-AGR-3 and p-AGR-4 were constructed for l-arginine overproduction. **b** l-arginine production and acetate formation of strain N11. **c** Cell growth of strain N11. N11: BW25113 *ΔargA ΔspeF ΔspeB ΔastA ΔargR*, overexpression of *argCBH* and *argDGI*. Error bars are the standard deviation for three independent experiments

Pulling external n-acetylglutamate flux towards l-ornithine is desired to obtain an efficient l-arginine overproducing strain. In the l-arginine biosynthesis pathway, gene *argB*, encoding n-acetylglutamate kinase, is responsible for the phosphorylation of n-acetylglutamate. The reductase ArgC encoded by gene *argC* catalyzes the reduction of n-acetylglutamyl-phosphate to generate n-acetylglutamate-5-semialdehyde, followed by amination (gene *argD* encoding n-acetylornithine aminotransferase) and deacylation (gene *argE* encoding acetylornithine deacetylase) to obtain l-ornithine. According to a previous report, the turnover number of gene *argE* is 3800 s^−1^ [45], indicating that enzyme ArgE has a high catalytic activity for converting n-acetylornithine to l-ornithine, and native gene *argE* expression is enough for l-arginine production. Therefore, plasmids overexpression of the *argCBH* and *argDGI* operons (p-AGR-3, p-AGR-4, shown in Fig. 5a) were constructed and co-transformed into strain N4, resulting in strain N11 (Table 1) for l-arginine biosynthesis.

The performance of strain N11 was evaluated in shake flask fermentation with 5 g/L n-acetylglutamate supply and was shown in Fig. 5. The OD_600_ of strain N11 reached 18.2 at the end of fermentation (Fig. 5c), but the titer of l-arginine was lower and only 1.1 g/L (Fig. 5b) was detected with a yield of 0.92 mol l-arginine/mol n-acetylglutamate (row #6 and column #5 in Table 2). The concentration of n-acetylglutamate in the broth remained at a level of 3.6 g/L. The major byproduct detected was acetate at 2.7 g/L during the fermentation due to overflow metabolism. These results suggested that intracellular l-arginine accumulation can be achieved with external n-acetylglutamate. The strain N11, using n-acetylglutamate as a co-substrate, showed a lower titer of l-arginine compared with strains N8 or N9 that synthesized l-arginine from l-ornithine as a co-substrate (column #5 in Additional file 1: Table S1). However, the l-arginine yield of strain N11 is higher than that of strains N8 or N9 (0.92 for N11 vs 0.69 for N8, 0.76 for N9, column #7 in Additional file 1: Table S1). The low titer came from the lower conversion of n-acetylglutamate.

**Table 2:** Shake flask fermentations of strain N11 with different concentrations of yeast extract

Yeast extract (g/L)	OD_600_	l-arginine (g/L)	Acetate (g/L)	∆ n-acetylglutamate (g/L)	l-arginine yields	
					Y_P/S_^a^ (mol/mol)	Y_P/C_^b^ (g/OD_600_)
0	10.3 ± 0.4	2.0 ± 0.8	1.2 ± 0.1	2.4	0.9	0.19
1	13.3 ± 1	2.4 ± 0.5	6.9 ± 0.7	2.8	0.93	0.18
2	16.8 ± 0.7	4.4 ± 0.0	7 ± 1.2	4.9	0.99	0.26
4	19.5 ± 0.8	1.6 ± 0.3	3.8 ± 0.4	1.9	0.91	0.08
5	18.2 ± 1.1	1.1 ± 0.4	2.7 ± 0.4	1.3	0.92	0.06

N11: BW25113 *ΔargA ΔspeF ΔspeB ΔastA ΔargR,* overexpression of *argCBH* and *argDGI*

Data are indicated as means ± SD

^a^Y_P/S_: l-arginine production yield vs. substrate (mol l-arginine/mol consumed n-acetylglutamate)

^b^Y_P/C_: l-arginine production yield vs. biomass (g l-arginine /OD_600_)

### Optimization of medium components

We speculated that the lower conversion of n-acetylglutamate was affected by the yeast extract in the medium, which contains abundant nitrogen sources and various amino acids. To investigate the effect of yeast extract on l-arginine production, an alternative fermentation medium was applied for the existing strain N11 (see “Materials and Methods”). The shake-flask fermentations and analysis of products were conducted as described in Table 2. The highest titer of 4.4 g/L l-arginine was produced by fermentations containing 2 g/L yeast extract, and the OD_600_ reached 16.8 at the end of cultivation (Table 2). In comparison, with 1 g/L yeast extract or without yeast extract, the l-arginine production performed similarly at a lower titer of 2.4 g/L and 2 g/L (Table 2), respectively. This may be partially due to the significantly reduced cell growth. The OD_600_ for feeding 1 g/L yeast extract or without yeast extract are 1.3 × and 1.6 × lower (OD_600_, 13 or OD_600_, 10, Table 2), respectively [46]. Further increasing yeast extract to 4 g/L or 5 g/L, the OD_600_ were 19.5 and 18.2 (Table 2), respectively, but the amount of l-arginine detected dropped to 1.6 g/L and 1.1 g/L, respectively. These results confirmed our hypothesis that the altered yeast extract concentration would influence both cell growth and l-arginine production from n-acetylglutamate. The highest utilization efficiency of n-acetylglutamate towards l-arginine could be achieved when supplied with 2 g/L yeast extract for fermentation.

With the increasing l-arginine production from n-acetylglutamate, there was 7 g/L acetate detected in the culture supernatants of fermentation at the end of the cultivation. In addition to the carbon overflow of glucose, the deacetylation process of n-acetyl-l-ornithine to l-ornithine during l-arginine production inevitably produces equal amounts of acetate. In a previous report, the heterologous *argJ* gene was substituted for the *argE* gene to reduce the formation of byproduct acetate and increase the yield of l-citrulline synthesis [38]. Acetate is an abundant carbon source candidate in nature, especially, in industrial and agricultural wastewater. Recently several studies have reported that acetate could be used for acetyl-CoA generation in *E. coli* to enhance the production of acetyl-CoA derived chemicals [47, 48]. Therefore, further work could be done to construct an acetate-assimilating pathway in our strains to increase the carbon yield of both substrates.

In addition, we noticed that, with 2 g/L yeast extract added, the Y_P/C_ was achieved 0.26 (g l-arginine/OD_600_) and the Y_P/S_ of n-acetylglutamate to l-arginine was up to 99% of the theoretical maximum. At the end of fermentation, no other amino acids such as l-glutamate, l-ornithine, and l-citrulline were detected, implying that the metabolic flux from n-acetylglutamate towards to l-arginine biosynthetic pathway was efficient. These results indicated our approach of synthesizing l-arginine from n-acetylglutamate is feasible to overcome the low yield in the traditional l-arginine synthesis pathways. Notably, although cell growth was relatively hampered for the strains with 1 g/L yeast extract or without yeast extract, the Y_P/C_ reached 0.18 and 0.19, respectively, much higher than that with the addition of 4 g/L or 5 g/L yeast extract (0.08 and 0.06, respectively). While we use yeast extract to demonstrate the importance of medium for fermentation, in the future, we can further optimize the fermentation by direct evolution of cells growing in n-acetylglutamate, lowering input of yeast extract or adding other substrates, to promote cell growth and achieving high-yield production of l-arginine.

### Co-production of l-arginine and pyruvate

After successfully reinforcing the metabolic flux of the l-arginine biosynthesis pathway from n-acetylglutamate, we attempted to optimize the utilization of input carbon resources in the fermentation system. Compared with the single production process, a co-production strategy is more economically appealing for biotechnology, which can utilize substrates more efficiently, decrease the accumulation of by-products and make full use of the production capacity of cells [48–50]. A similar strategy has been employed for the co-production of 1,4-butanediol and mevalonate from glucose and d-xylose in engineered *E. coli* [51]. Here, we used pyruvate production as an example. Pyruvate is one of the essential chemicals generated in the fermentation process, as a potential precursor to various chemicals, which has versatile applications in the fields of pharmaceuticals, food, and agricultural chemicals [52].

**Fig. 6:**
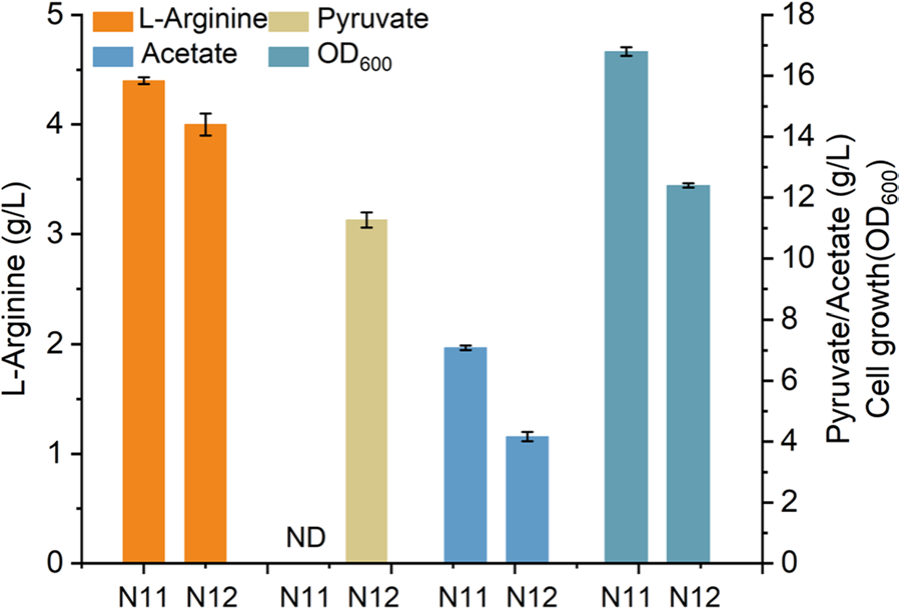
Comparison of l-arginine, pyruvate, acetate titers, and the cell growth in strains N11 and N12. N12: BW25113 *ΔargA ΔspeF ΔspeB ΔastA ΔargR*, *ΔldhA ΔadhE ΔaceE ΔpoxB ΔpflB,* overexpression of *argCBH* and *argDGI*. Error bars are the standard deviation for three independent experiments

To achieve the goal of co-production of l-arginine and pyruvate, genes involved in ethanol, lactic acid, acetate, and formate formation (*adhE, ldhA, poxB, pflB, and aceE*) in N11 were removed to increase pyruvate accumulation [53], resulting in strain N12 (Table 1). With a consumption of 40 g/L glucose and 5 g/L n-acetylglutamate, the strain N12 was shown to accumulate 4 g/L l-arginine and 11.3 g/L pyruvate in shaking fermentation (column #2 and column #4 in Fig. 6). However, in the fermentation of strain N11, there was no pyruvate detected, which demonstrated that our strain N12 can achieve a significant increase of pyruvate accumulation. There was a slight decrease in the production of l-arginine compared to N11 (4.4 g/L for N11 vs 4 g/L for N12, column #1 and column #2 in Fig. 6), probably due to the lower biomass (OD_600_, 12.4 vs 16.8, column #8 and column #7 in Fig. 6). The impaired growth of N12 may be affected by the reduced carbon flux towards the TCA cycle [54]. It is necessary to promote better cell growth by further metabolic optimization in the future to increase the l-arginine production capacity of cells. Besides, the acetate secretion was reduced compared with strain N11 (7 g/L for strain N11 vs 4.2 g/L for strain N12, column #5 and column #6 in Fig. 6), which may be attributed to the knocking out of gene *poxB* [55]. These results have validated that the co-production strategy of l-arginine and pyruvate is feasible in the strain N12 we constructed. In addition, the global average manufacturer selling price for pyruvate and glucose was ~$10/kg and $0.8/kg, respectively [56, 57], which means that this strategy is also economically promising. Pyruvate yield can be improved in the future by adaptive evolution [58].

## Conclusions

In this study, we established a novel approach for the high-yield production of l-arginine by directly feeding key precursor n-acetylglutamate. Firstly, gene *argA* was removed from BW25113 to construct an l-arginine auxotroph and disable n-acetylglutamate endogenous biosynthesis. The feasibility of external n-acetylglutamate towards l-arginine was verified via growth assay in *argA*^*−*^ strain. Secondly, to achieve the accumulation of l-arginine, genes *speF, speB, astA, and argR* were removed from the *argA*^*−*^ strain to block the l-arginine degradation pathway and the feedback regulation, respectively. Based on overexpression of *argDGI* and *argCBH* operons*,* the strain N11 could produce ~ 4 g/L l-arginine with 5 g/L of n-acetylglutamate supplied in shaking flask fermentation. The carbon yield of n-acetylglutamate towards l-arginine was up to 99% of the theoretical maximum (0.99 mol l-arginine/mol n-acetylglutamate). Finally, to avoid the waste of glucose cosubstrate, a co-production strain N12 was constructed and achieved the integrated production of l-arginine and pyruvate with a titer of 4 g/L and 11.3 g/L, respectively. In conclusion, our results realize the high yield of l-arginine production from n-acetylglutamate precursor and provide another competitive possibility for improving the metabolic flux yield in the l-arginine biosynthesis pathway. In the future, we can further adjust and optimize the metabolic flux to maximize the utilization of both substrates.

## Materials and methods

### Strains and media

The *E. coli* strains used in this study are listed in Table 1. The fermentation strains were derived from the wild-type *E. coli* K-12 strain BW25113. The *E. coli* DH5α was used as the cloning host for plasmid construction. Unless otherwise specified, all strains were cultivated at 37 ℃ in LB media (10 g/L tryptone, 5 g/L yeast extract, and 10 g/L sodium chloride) medium with appropriate antibiotics.

### Knocking out chromosomal genes

Gene deletion was performed using P1 transduction and P1 phages of *argA, argR, speF, speB*, *astA, ldhA, adhE, aceE, pflB,* and *poxB* were obtained from the Keio collection [59]. Colonies containing the correct deletions were transformed with plasmid pCP20 to remove the kanamycin resistance marker at 30 ℃. The correct knockouts were verified by colony PCR. Gene *argA* was deleted from the *E. coli* strain BW25113 chromosome to make an l-arginine auxotroph.

### Growth assay

To assess the essentiality of n-acetylglutamate for the l-arginine auxotrophic strain growth, the growth assays of *E. coli ΔargA* strain were conducted in three different mediums: M9 minimal medium containing 5 g/L glucose, M9 minimal medium containing 5 g/L glucose with supplement of 5 g/L l-arginine, and M9 minimal medium containing 5 g/L glucose with supplement of varying levels of n-acetylglutamate (0 g/L, 1 g/L, 5 g/L, 10 g/L, 20 g/L). These media do not contain yeast extract. Three fresh colonies of *E. coli ΔargA* strain were inoculated overnight in LB, and the resulting cells were washed twice with 2 mL of ice-cold sterile deionized water and suspended again. 20 μL of cultures were transferred into 2 mL of three different minimal media, respectively. The cell growth (optical density) was measured every few hours. OD of all strains was measured using a spectrophotometer at 600 nm, and the cell densities were normalized before starting the assays.

### Plasmids construction

Primers (Table 3) were ordered from Tsingke. PCR reactions were carried out with Q5 High-Fidelity DNA polymerase according to the manufacturer's instructions. The sequences of all the plasmids produced were verified by DNA sequencing. The details for all strains and plasmids are shown in Table 1. A gene fragment encoding lac repressor LacI [60] was inserted into the EcoRI site of plasmid pZE12 and pZA24 [61] to yield plasmid pZElac with ampicillin resistance, and pZAlac with kanamycin resistance, respectively.

**Table 3:** Primers used in this study

Primers	Sequence (5′ to 3′)
argI-F-1	TTAAAGAGGAGAAAGGTACCATGTCCGGGTTTTATCATAAGCATTTC
argI-R-1	CGTCATAGTTAATTTCTCCTACTAGTTTATTTACTGAGCGTCGCGACCAT
argG-F-1	CTAGTAGGAGAAATTAACTATGACGACGATTCTCAAGCATCTCCC
argG-R-1	GCCATAGTTAATTTCTCCTAAGCTTTTACTGGCCTTTGTTTTCCAGATTCTC
argH-F-1	CAGTAAAAGCTTAGGAGAAATTAACTATGGCACTTTGGGGCGGGCGTTTTAC
argH-R-1	TTATTTGATGCCTCTAGATTACCCTAACCGAGCCTGCGCAAAAG
carAB-F-1	TTCATTAAAGAGGAGAAAGGTACCATGATTAAGTCAGCGCTATT
carAB-R-1	TAATTTCTCCTTCTAGATTATTTGATCTGTGCGTGCATTTC
arg-CBH-F-1	AAGAGGAGAAAGGTACCATGTTGAATACGCTGATTGTGGGTG
arg-CBH-R-1	CTTTCGTTTTATTTGATGCCTCTAGATTACCCTAACCGAGCCTGCGCAAAAG
argD-F-1	TTCATTAAAGAGGAGAAAGGTACCATGGCAATTGAACAAACAGCAATTACA
argD-R-1	GTCGTCATAGTTAATTTCTCCTAAGCTTTTACGCCCCAACCACCTTCGCCACC
argG-F-2	AGAAATTAACTATGACGACGATTCTCAAGCATCTCCCGGTAGGTCAACG
argG-R-2	TAGTTAATTTCTCCTACTAGTTTACTGGCCTTTGTTTTCCAGAT
argI-F-2	GGCCAGTAAACTAGTAGGAGAAATTAACTATGTCCGGGTTTTATCATAAGCA
argI-R-2	TTTGATGCCTCTAGAGCTAGCTTATTTACTGAGCGTCGCGACCATCA
Del-argA-F	CGAATAATCATGCAAAGAGGTGTGCCATGGTGTAGGCTGGAGCTGCTTC
Del-argA-R	GTGTTACGCATGTCGCATCCGACGATTTTCATCGCTTACCCTAAATCCGCCAT
Del-argR-F	ATCAACCACCATATCGGGTGACTTATGGTGTAGGCTGGAGCTGCTTC
Del-argR-R	GTCAGAAACGACGGGGCAGAGATTAAAGCTCCTGGTCGAACAGCTCT
Del-astA-F	GCTGCTTGCGAACACTTTGTTAGCCGAGGTTCATCATGGTGTAGGCTGGAGC
Del-astA-R	CTCTGCGCACAGGCGCACCAGACGAACATTCCGGGGATCCGTCGACC
Del-speF-F	TGAGGACCTGCTATTACCTAAAATAAAGAGATGAAAAATGGTGTAGGCTGG
Del-speF-R	ATTTTTCCCCTTTCAACAGGGTGCTTTGATTCCGGGGATCCGTCGACC
Del-speB-F	TTTTTTATATCGACTTTGTAATAGGAGTCCATCCATGGTGTAGGCTGGAGCTG
Del-speB-R	CCCTTTTTCGCCGCCTGAATATACAGATTCCGGGGATCCGTCGACC

**Table:** 

pZE-* P*_*LlacO1*_*-argI-argG-argH* Genes *argI, argG,* and *argH* were amplified based on *E. coli* genomic DNA. Primers argI-F-1 and argI-R-1 were used to amplify gene *argI*. Primers argG-F-1 and argG-R-1 were used to amplify gene *argG,* and primers argH-F-1 and argH-R-1 were used to amplify gene *argH*. Then these three fragments and the vector fragment of pZElac were homologous recombined with Exnase to form plasmid pZE-P_*LlacO1*_-*argI-argG-argH*
pZE-*P*_*LlacO1*_*-carAB-argI-argG-argH* Genes *carAB, argI, argG,* and *argH* were amplified based on *E. coli* genomic DNA. Primers carAB-F-1 and carAB-R-1 were used to amplify gene *carAB*. Primers argI-F-1 and argI-R-1 were used to amplify gene *argI*. Primers argG-F-1 and argG-R-1 were used to amplify gene *argG,* and primers argH-F-1 and argH-R-1 were used to amplify gene *argH*. Then these four fragments and the vector fragment of pZElac were homologous recombined with Exnase to form plasmid pZE-P_*LlacO1*_- *carAB-argI-argG-argH*
pZE-*P*_*LlacO1*_-*argCBH* Gene cluster *argCBH* was amplified based on *E. coli* genomic DNA. Primers arg-CBH-F-1 and arg-CBH-R-1 were used to amplify genes *argCBH*. Then this fragment and the vector fragment of pZElac were homologous recombined with Exnase to form plasmid *pZE-P*_*LlacO1*_*-argCBH*
pZA-*P*_*LlacO1*_-*argD-argG-argI* Genes *argD, argG,* and *argI* were amplified based on *E. coli* genomic DNA. Primers argD-F-1 and argD-R-1 were used to amplify gene *argD*. Primers argI-F-2 and argI-R-2 were used to amplify gene *argI*. Primers argG-F-2 and argG-R-2 were used to amplify gene *argG*. Then these three fragments and the vector fragment of pZAlac were homologous recombined with Exnase to form plasmid pZA-P_*LlacO1*_*-argD-argG-argI*

### Shake flask fermentation

Unless otherwise specified, all the strains were incubated in the fermentation medium (10 mL M9 minimal media supplemented with 5 g/L yeast extract, 40 g/L glucose, 5 mM coenzyme B12, 5 g/L n-acetylglutamate. Antibiotics were added appropriately (100 mg/L ampicillin, 100 mg/L Kanamycin). To explore the feasibility of l-arginine production from l-ornithine, 10 g/L l-ornithine was supplied in the fermentation of strains BW25113 and N5-N10 (without n-acetylglutamate). To explore the effects of yeast extract on l-arginine production, different concentrations (0, 1, 2, 4, and 5 g/L) of yeast extract were supplemented in the fermentation of strain N11. 200 μL of overnight cultures were transferred into fermentation medium in 150 mL conical flasks. Isopropyl-β-D-thiogalactoside (IPTG) was added at a final concentration of 1 mM to induce the overexpression of introduced genes for the production of l-arginine. The culture broth was buffered by 0.5 g CaCO_3_. Fermentation was carried out in a shake incubator at 250 rpm at 30 ℃. Fermentation products were analyzed via high performance liquid chromatography (HPLC) and error bars represent the standard deviation from three independent experiments, by picking three independent colonies for fermentation.

### Measurement of optical density and metabolites analysis

Fermentation samples were collected at 24-h intervals. For measurement of the optical density at 600 nm (OD_600_), 100 μL of the sample was mixed with 900 μL of 1 M HCl to eliminate the CaCO_3_ in the fermentation medium. Metabolites were analyzed using an Agilent 1260 Infinity HPLC system. The concentrations of n-acetylglutamate, acetate, and glucose were analyzed using an Aminex HPX 87 H column (Bio-Rad, USA) and a refractive-index detector. The mobile phase is 5 mM H_2_SO_4_ with a flow rate of 0.6 mL/min. The column temperature and detection temperature are 35 ℃ and 50 ℃, respectively. The concentrations of l-arginine, l-glutamate, and l-ornithine were analyzed using Agilent C18 column (4.6 × 100 mm, 3.5 mm) and a DAD detector. The mobile phase gradient program and automated liquid sampler program were performed as the manufacturer's instruction (http://www.chem.agilent.com/Library/applications/5990-4547EN.pdf).

### Chemical conversion of l-glutamate to n-acetylglutamate

The n-acetylglutamate was prepared as described in previous research [24] and details were shown in Supplementary materials (Additional file 1: Figs. S1, S2). n-acetylglutamate was synthesized efficiently as the precursor for the production of l-arginine in *E. coli* by shaking flask fermentation.

### Supplementary Information

**Additional file 1: Table S1.** Comparison of l-arginine production in strains N8, N9 and N11. **Figure S1.** n-acetylglutamate was synthesized by chemical acetylation of l-glutamate with acetic anhydride. **Figure S2.** HPLC signal shows n-acetylglutamate accumulation.

## Data Availability

All data generated or analyzed during this study are included in this published article and its additional files.

## Abbreviations

CPS: Carbamoyl phosphate
DHAP: Dihydroxyacetone phosphate
F6P: Fructose 6-phosphate
G3P: Glyceraldehyde 3-phosphate
G6P: Glucose 6-phosphate
N-acetyl-P: N-acetylglutamate-phosphate
N-acetyl-sa: N-acetylglutamate-semialdehyde
