# Correction: A novel strategy for L-arginine production in engineered *Escherichia coli*

**DOI:** 10.1186/s12934-024-02328-x

**Published:** 2024-02-10

**Authors:** Mengzhen Nie, Jingyu Wang, Kechun Zhang

**Affiliations:** 1 Zhejiang University, Hangzhou 310027 Zhejiang, China; 2 Center of Synthetic Biology and Integrated Bioengineering, School of Engineering, Westlake University, Hangzhou 310030 Zhejiang, China

**Correction to: Microbial Cell Factories (2023) 22:138**

10.1186/s12934-023-02145-8

In the Funding section of this article [1] the grant number relating to National Natural Science Foundation of China was incorrectly given as 10327A012001 and should have been 22078267. The original article has been corrected.
