# Supplementary material for: A novel strategy for l-arginine production in engineered Escherichia coli
Source: Microb Cell Fact. 2023 Jul 26;22:138. doi: 10.1186/s12934-023-02145-8 (PMC10373293; doi:10.1186/s12934-023-02145-8)
Supplement: Supplementary file 1 — Additional file 1: Table S1. Comparison of l-arginine production in strains N8, N9 and N11. Figure S1. n-acetylglutamate was synthesized by chemical acetylation of l-glutamate with acetic anhydride. Figure S2. HPLC signal shows n-acetylglutamate accumulation. [file 12934_2023_2145_MOESM1_ESM.docx]

**Additional file**

**A novel strategy for L-arginine production in engineered *Escherichia coli***

Mengzhen Nie^1,2^, Jingyu Wang^2^, Kechun Zhang^2 *^

^1^ Zhejiang University, Hangzhou, Zhejiang 310027, China

^2^ Center of Synthetic Biology and Integrated Bioengineering, School of Engineering, Westlake University, Hangzhou, Zhejiang 310030, China

**Table S1** Comparison of L-arginine production in strains N8, N9 and N11.

| **strains** | **subtrates** | **overexpression genes** | **OD_600_** | **L-arginine**  **(g/L)** | **Acetate**  **(g/L)** | **L-arginine**  **yield (mol/mol)^a^** |  |  |
| --- | --- | --- | --- | --- | --- | --- | --- | --- |
| N8 | glucose+  L-ornithine | *argIGH* | 13±1 | 6.7±0.2 | 2.5±0.5 | 0.69 |  |  |
| N9 | glucose+  L-ornithine | *argIGH,*  *carAB* | 13.3±0.4 | 7.6±0.1 | 2.3±0.4 | 0.76 |  |  |
| N11 | glucose+  N-acetylglutamate | *argCBH*  *argDGI* | 18.2±1.1 | 1.1±0.4 | 2.7±0.4 | 0.92 |  |  |

^a^ L-arginine yield: L-arginine production (mol)/consumed substrate (mol). (For strains N8 and N9, the substrate represents L-ornithine; for strain N11, the substrate represents N-acetylglutamate.)

**Fig. S1** N-acetylglutamate was synthesized by chemical acetylation of L-glutamate with acetic anhydride. The sodium L-glutamate (l g, 8 mmol), water (8 mL), and acetic anhydride (1.2 mL) were taken in a beaker (50 mL) and sonicated for 10 mins at ambient temperature. At the end of the reaction, the N-acetylglutamate concentration of samples was analyzed using HPLC. The water and acetic acid were removed completely and collected at 50℃ on a rotary vacuum evaporator.


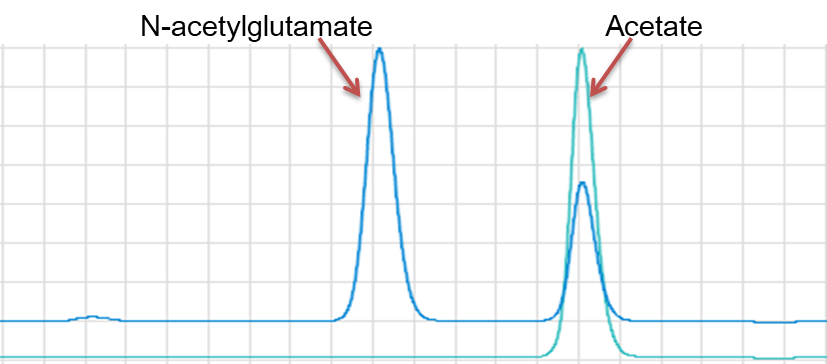


**Fig. S2** HPLC signal shows N-acetylglutamate accumulation.
